# Whole Ovine Ovaries as a Model for Human: Perfusion with Cryoprotectants *In Vivo* and *In Vitro*

**DOI:** 10.1155/2014/409019

**Published:** 2014-02-19

**Authors:** Vladimir Isachenko, Gohar Rahimi, Maria Dattena, Peter Mallmann, Saltanat Baikoshkarova, Elisabeth Kellerwessel, Marat Otarbaev, Tamara Shalakhmetova, Evgenia Isachenko

**Affiliations:** ^1^Department of Obstetrics and Gynecology, Cologne University, Kerpener Stra**β**e 34, 50931 Cologne, Germany; ^2^Agris-Sardegna, DIRPA, Reproduction Division, S.S. 291 Km, 7100 Sassari, Italy; ^3^Faculty of Biology and Biotechnology, Al-Farabi Kazakh National University, Prospekt Al-Farabi 71, Almaty 50040, Kazakhstan; ^4^Teraerzte-Team K-Suelz, Rennebergstra**β**e 23, 50939 Cologne, Germany

## Abstract

These experiments were performed to test the perfusion of ovine as a model for human ovaries by cryoprotectants *in vivo* at high temperature when the permeability of capillaries is high and when blood is insensibly replaced by the solution of cryoprotectants. By our hypothetical supposition, ovaries could be saturated by cryoprotectants before their surgical removal. The objective was to examine the effectiveness of perfusion of ovine ovaries with vascular pedicle *in vivo* and *in vitro*. *Arteria ovarica* was cannuled and ovaries were perfused by Leibovitz L-15 medium + 100 IU/mL heparin + 5% bovine calf serum + 6% dimethyl sulfoxide + 6% ethylene glycol + 0.15 M sucrose + Indian ink *in vivo* and *in vitro*. In the first and second cycle of experiments, ovaries (*n* = 13 and *n* = 23) were perfused *in vivo* and *in vitro*, respectively, during 60 min with the rate of perfusion 50 mL/h (0.8 mL/min). It was established with *in vivo* perfusion that only about 10% of ovarian tissues were perfused due to an appearance of multiple anastomoses when the perfusion medium goes from *arteria ovarica* to *arteria uterina* without inflow into the ovaries. It was concluded that *in vitro* perfusion of ovine intact ovaries with vascular pedicle by freezing medium is more effective than this manipulation performed *in vivo*.

## 1. Introduction

Cryopreservation of whole ovine ovaries with vascular pedicles is an important focal point of research in medicine when ovine ovary is an animal model for human ovary.

In medicine, due to the increasing effectiveness of cancer treatments and good long-term prognosis for young women, the problem of postcancer infertility plays an important role. It is because chemotherapy, depending on the treatment regime chosen, can be gonadotoxic and lead to the functional death of the ovaries. The cryopreservation of ovarian tissue before cancer therapy with retransplantation after convalescence is the key to solving this problem [1–3].

In a recent publication of a research group from France, which is one of the leaders of cryopreservation of whole mammalian ovaries [4], it is noted that cryoprotectant diffusion into perfused ovine ovaries is a potentially limiting factor that has not been adequately investigated [4].

Cryopreservation and transplantation of whole ovaries with vascular pedicle would be helpful to prevent posttransplantation ischemia. However, the data have been reported as evidence that after cryopreservation of whole ovine ovaries their viability is low [5, 6].

Development of technology of long-time perfusion of intact ovaries by cryoprotectants at low temperatures is important because earlier it was established that 24 h cooling to 5°C before cryopreservation is beneficial for freezing human ovarian fragments [7]. It was noted that the quality of follicles and the intensiveness of neovascularisation observed in ovarian tissue precooled before culture and precooled before cryopreservation were drastically increased [7].

These experiments were performed to test the perfusion of human ovaries by cryoprotectants *in vivo* at high temperature when the permeability of capillaries is high and when blood will be insensibly replaced by the solution of cryoprotectants. Our speculation for performing experiments was the following: *in vivo *perfusion of ovaries by cryoprotectants before their removal and freezing can be more successful in comparison with this procedure *in vitro*. By our hypothetical supposition, ovaries could be saturated by cryoprotectants before their surgical removal.

The aim of this research was to study the effectiveness of perfusion of whole ovine ovaries with vascular pedicle *in vivo* and *in vitro*.

## 2. Materials and Methods

Except where otherwise stated, all chemicals were obtained from Sigma (Sigma Chemical Co., St. Louis, MO, USA).

Our experiments were performed under a protocol approved by the University Ethics Board. It was used for the ovaries from Sarda animals with the body weight from 44 to 51 kg.

Freezing medium included Leibovitz L-15 medium + 100 IU/mL heparin + 5% bovine calf serum + 6% dimethyl sulfoxide (DMSO) + 6% ethylene glycol + 0.15 M sucrose + 20% Indian ink. Experimental perfusions were performed during 60 min with the rate of perfusion 50 mL/h (0.8 mL/min).

### 2.1. *In Vivo* Perfusion

**Figure 1:**
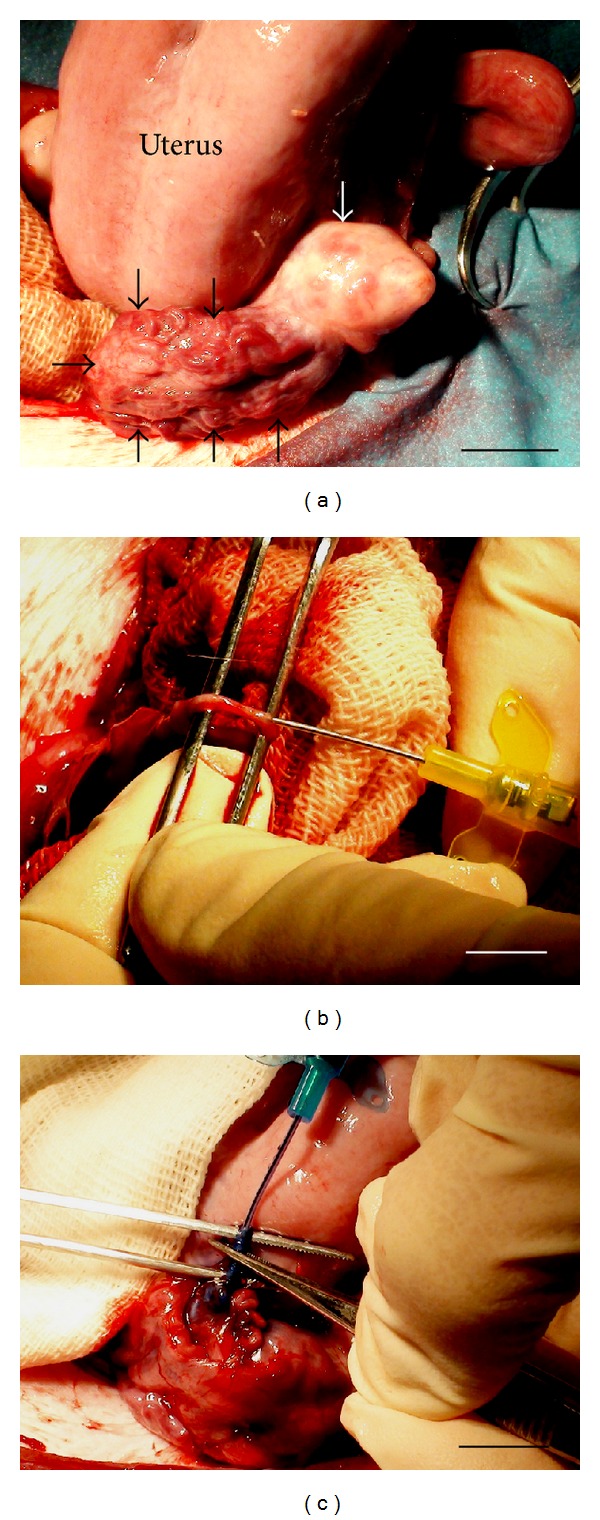
*In vivo* perfusion of ovine intact ovary with freezing medium. (a) Expelling of ovary (white arrow) with pedicle (black arrow) to operation field, (b) perforation of* arteria ovarica *by 24 G catheter for perfusion with freezing medium, and (c) beginning of perfusion of ovary with freezing medium through *arteria ovarica *with 18 G catheter. Bar = 1 cm.

Ovary (*n* = 13) with pedicle was expelled to operation field (Figure 1(a)). Then *arteria ovarica* was perforated by the needle of 24 G catheter and then 18 G catheter (Introcan Safety, B. Braun Melsungen AG, Melsungen, Germany) was fixed for perfusion with freezing medium (Figure 1(b)). *In vivo* perfusion was performed at 35°C (because ovary and pedicle are partially located inside of the body) (Figure 1(c)). The temperature of freezing medium was 37°C. Fallopian tubes were not separated, mesovarium was not removed, and uteroovarian anastomosis was not ligated.

### 2.2. *In Vitro* Perfusion

**Figure 2:**
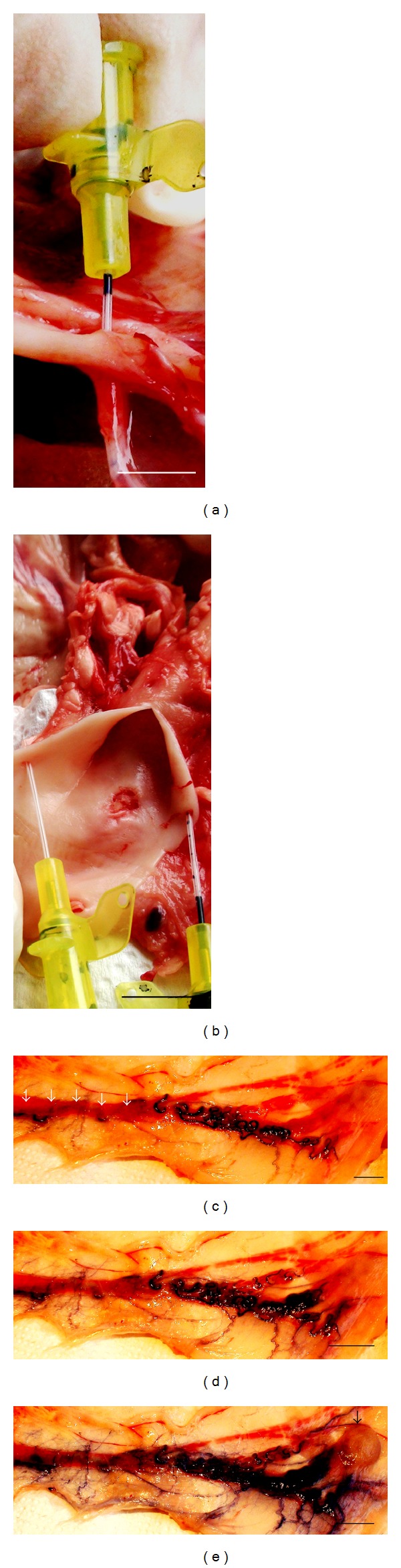
*In vitro* perfusion of ovine intact ovary with freezing medium. ((a), (b)) *Aorta *with *arteria ovarica *(24 G catheters inside) and ((c), (d), and (e)) perfusion with freezing medium: 3 min (c), 5 min (d), and 10 min (e) elapsed from the beginning of perfusion, *vena ovarica *(white arrows), ovary (black arrow). Freezing medium includes Leibovitz L-15 medium + 100 IU/mL heparin + 5% bovine calf serum + 6% dimethyl sulfoxide + 6% ethylene glycol + 0.15 M sucrose + Indian ink. Bar = 1 cm.

The conditions of our experiments allowed obtaining ovaries (*n* = 23) with pedicles for *in vitro* perfusion during 3–5 min after the slaughtering of animals. The 24 G catheter (B. Braun) through *aorta* was introduced to *arteria ovarica *and fixed inside (Figures 2(a) and 2(b)). *In vitro *perfusion was performed at room temperature (22°C).

**Figure 3:**
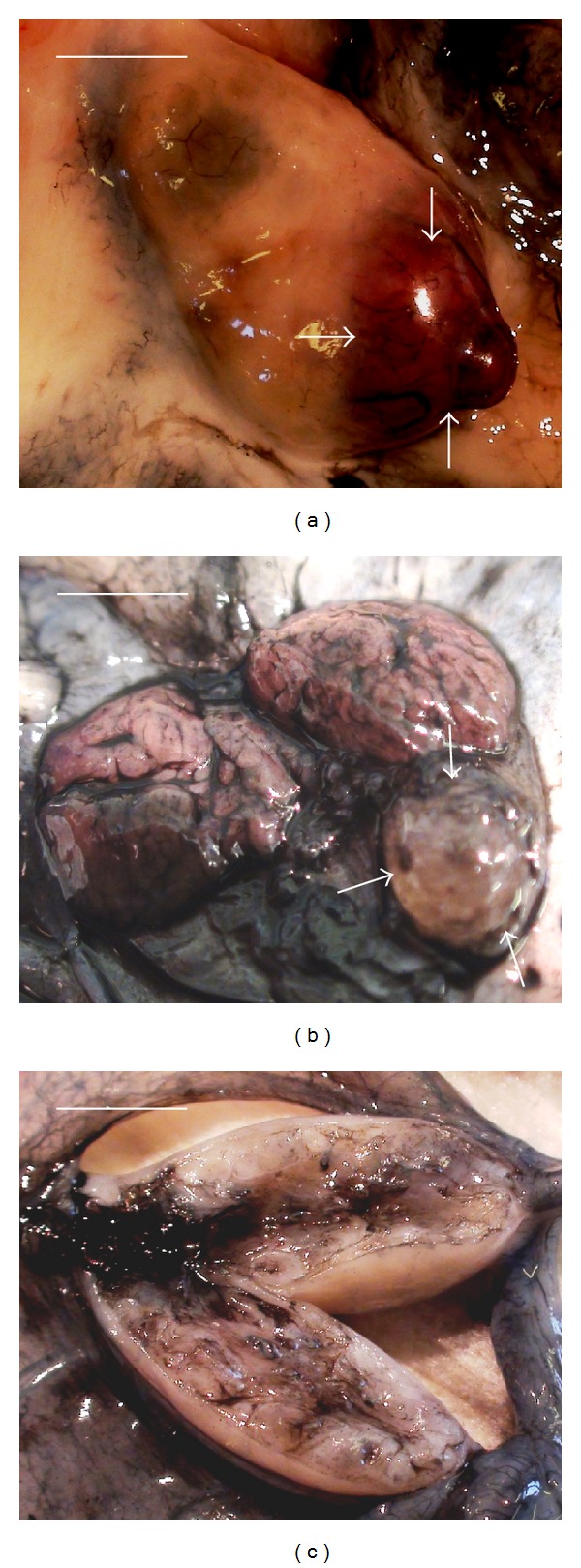
End of perfusion of ovine intact ovary with vascular pedicle with freezing medium. ((a), (b)) Ovary after successful perfusion (~95% of tissues excluding yellow body) and (c) ovary after unsuccessful perfusion (~10% of tissues), yellow body (arrows). Note the unsuccessful perfusion of yellow body. Freezing medium includes Leibovitz L-15 medium + 100 IU/mL heparin + 5% bovine calf serum + 6% dimethyl sulfoxide + 6% ethylene glycol + 0.15 M sucrose + Indian ink. Bar = 0.5 cm.

To the end of the experiments, after 60 min of perfusion, the success rate of perfusion of ovaries was detected with analysis of images by the percentage of Indian-ink-perfused tissues (Figure 3). Perfusion was denoted as successful if about 100% of ovarian tissues were perfused by Indian ink.

Effectiveness of perfusion was evaluated by ANOVA. The level of statistical significance was set at a *P* < 0.05.

## 3. Results and Discussion

It was established that *in vivo* perfusion can be evaluated as unsuccessful; only about 10% of ovarian tissue was perfused (Figure 3) due to an appearance of anastomoses between *arteria ovarica *and *arteria uterina *when the perfusion medium goes from *arteria ovarica* to *arteria uterina* without inflow into the ovaries. It was technically difficult and practically not possible to close mechanically these multiple anastomoses during the perfusion.

After successful perfusion, approximately 100% of ovarian tissue and its vascular pedicle obtained blue colour (Figures 2 and 3).

### 3.1. Aim of Perfusion and Importance of Postthawing Storage of Capillaries

We believe that the aim of ovarian perfusion before cryopreservation is not saturation of cells by permeable cryoprotectants but the expelling of blood cells from blood vessels. In our opinion, if ovarian blood vessels and especially capillaries will be filled with coagulated blood cells before freezing, restoration of normal blood circulation in ovarian tissue after thawing will be not possible.

These blood cells cannot be frozen in vessels, together with ovarian tissue, without prefreezing separation. Technologically it is difficult to do it. Ovarian tissue and blood cells must be frozen with different methodologies, so that we have only one possibility to avoid ischemia due to the presence of blood cells in vessels: to expel these cells from vessels before the cooling of ovarian tissue.

We chose the freezing medium for perfusion of ovaries because this freezing medium includes DMSO, liquid with very high rate of permeability. Presence of DMSO and heparin in the perfusion medium increases the ability of the freezing medium to evacuate blood cells from vessels before the freezing. Additionally, the saturation of ovarian tissue by permeable cryoprotectants was the second aim of perfusion of this tissue with freezing medium.

It was recognized that the presence of blood vessels is a very important ingredient for the successful ovarian tissue transplantation, and the establishment of the blood supplies is crucial for the survival of ovarian follicles [8]. It was demonstrated that transplanted immature rat ovaries become profusely revascularised within 48 h after autotransplantation [8]. In some opinions, in the cortex, development of primordial follicles is fully dependent on stromal vessels [9]. Prior to revascularisation, implants are vulnerable to ischemia, which is the primary obstruction to the survival of tissue after transplantation. Such damage can lead to a 30 to 70% decrease in graft size accompanied with fibrotic changes [10]. The hypoxia observed during the first 5 days after grafting and ischemic damage occurring during this period could induce primordial follicle loss [11–13] and disorders of follicular activation [14, 15].

In contrast with transplantation of cortex and transplantation of intact ovaries with pedicles, adequate perfusion can indirectly eliminate ischemia after postthawing transplantation.

During cryopreservation of whole ovaries with postthawing retransplantation through vascular anastomosis, the problem of neovascularisation will be solved “automatically,” because “old” capillaries begin to function just after connection of *arteria ovarica* with the circulatory system.

The data about the successful transplantation of human intact ovary with its vascular pedicle after cryopreservation are absent. However, cryopreservation of whole ovaries with vascular anastomosis with postthawing retransplantation can be viewed as a promising strategy for cancer patients. Some reports have described fresh (nonfrozen) whole ovaries with vascular anastomosis in human [16–18], and one pregnancy was observed [19].

### 3.2. Our Hypothetical Supposition for Performing of These Experiments

The following hypothetical supposition was the ground for performing of our comparative experiments. We did presuppose that blood which goes through the capillaries of ovarian tissue will be removed from these capillaries more effectively *in vivo* than *in vitro.* We guessed that, if we connect *arteria ovarica* through perfusor with cryopreservation medium, it will create the stable increasing pressure of this medium, and then blood slowly will be replaced by freezing medium. After isolation of *arteria ovarica* and *vena ovarica* from circulating system, we will obtain ovary that will be free from blood and ready for freezing. Likewise, we have presupposed that we will perform our perfusion manipulations *in vivo* when normal physiological negative pressure in the ovary from site of *vena ovarica* is observed. Theoretically, by *in vivo* perfusion of ovary the herd can play the role of additional pump for perfusion solution: this fact will help perfusion medium to penetrate through the capillaries of the ovary.

### 3.3. Cryopreservation of Whole Animal Ovaries at Present

The problem of cryopreservation of intact ovary with its vascular pedicle in animals is likewise far from solving.

Despite numerous attempts and the evidence of restoration of long-time follicular development [5, 6, 20–29], only three births after retransplantation of frozen ovine ovaries were noted.

The important role of perfusion of whole ewe ovaries by cryoprotective medium was recently demonstrated [4]. An observational study of 360 ewe ovaries stained by *in vitro* perfusion with qualitative marker of tissue blood supply was performed. A logistic regression model was established to identify the genes associated with incomplete ovary staining. Whole ewe ovaries with their vascular pedicles were perfused at 0.35 mL/min for 2 h at 39°C under 19 experimental conditions. The pedicles were removed and the ovaries cut and photographed. The unstained area of the slice surface was measured. It was noted that unstained areas were observed in 64.4% of the ovaries. Multivariate analysis found that incomplete ovary staining was independently associated with the lower experimenter experience, smaller ovary slice surface area, and the presence of a *corpus luteum*. The presence of unstained areas was independent of experimental conditions. The effectiveness of ovarian perfusion decreased from 83 to 60% [4]. The formulated conclusion of these experiments is that blood-supply impairments that result in incomplete perfusion might adversely affect outcomes after whole ovary cryopreservation. Improved perfusion techniques should enhance success [4].

In experiments of Gerritse et al. [30] the Indian ink perfusion studies were performed on bovine intact ovaries. The authors noticed that especially the larger vessels were well perfused, whereas the smaller vessels and the capillaries were less well perfused. The authors assume that the relatively smaller molecular weights of cryoprotective agents such as dimethyl sulfoxide and sucrose imply that they would be less subject to molecular filtering mechanisms of endothelial cells and basal membranes [30].

The presence of cryoprotectants (especially nonpermeable cryoprotectant sucrose) and bovine calf serum in the perfusion solution can explain that for the successful perfusion of small blood vessels and especially capillaries we need the speed of the perfusion medium slower than in experiments of Gerritse et al. [30] (2.5 mL/min).

### 3.4. DMSO in Perfusion Solution

Our freezing medium includes 6% DMSO and we used this cryoprotectant at 35–37°C.

What could happen when ovarian tissue will be perfused and later frozen with DMSO? Is it dangerous? To answer this question, we can image another object, which is more cryosensitive than ovarian tissue: human oocytes.

Earlier we performed a series of experiments to look into the role of DMSO during the cryopreservation by direct plunging into liquid nitrogen (vitrification) of human GV-oocytes. In one experiment, we studied the possibility of using a vitrification solution that was completely free of DMSO [31] because DMSO reportedly affects the organization of microfilaments in mouse oocytes [32] and induces chromosomal abnormalities (i.e., increases in the rates of degeneration and polyploid embryos) after cryopreservation of mouse oocytes in the presence of DMSO [33]. However, in our experiments, the absence of DMSO in the vitrification medium caused a significant decrease in maturation rates. On the other hand, a 5-minute contact of oocytes with DMSO at 37°C was enough to cause spontaneous oocyte activation and parthenogenetic development [31]. However, we have found the following solution of the problem. The protocol, which we recommend for use in medical practice, is a compromise between the presence of DMSO in vitrification medium and parthenogenetic activation. On the basis of our results, we propose (1) short (1-minute) contact with DMSO, which is only included in the last solution of vitrification medium, and (2) contact with DMSO at room temperature, which can theoretically decrease the negative effect on oocytes. The high maturation rates and the absence of parthenogenesis are a direct proof that this way of using DMSO (decreasing time and temperature of contact) is suitable for vitrification of human GV-oocytes.

On what ground do we have to say that 6% DMSO in perfusion medium (and later in freezing mediums) is not toxic for ovarian tissue?

Firstly, toxicity of this cryoprotectant is brightly discussed in cases of vitrification of oocytes and embryos, when concentration is up to 20%. Our perfusion and freezing solution includes only 6% DMSO. In medical practice DMSO in such concentration is brightly using for cryopreservation of a number of different tissues [7, 34–43].

Secondly, the potential toxic effect of DMSO can be compensated by detergent properties of DMSO, due to the presence of which the permeability of the perfusion medium through small capillaries will be increased.

## 4. Conclusions

In conclusion, *in vitro* perfusion of ovine whole ovaries with vascular pedicle by freezing medium is more effective than this manipulation performed *in vivo.*
